# Comparison of Liquid-Based Cytology and Conventional Papanicolaou Smear for Cervical Cancer Screening: An Experience From Pakistan

**DOI:** 10.7759/cureus.12293

**Published:** 2020-12-26

**Authors:** Atif A Hashmi, Samreen Naz, Omer Ahmed, Syed Rafay Yaqeen, Muhammad Irfan, Muhammad Ghani Asif, Anwar Kamal, Naveen Faridi

**Affiliations:** 1 Pathology, Liaquat National Hospital and Medical College, Karachi, PAK; 2 Internal Medicine, Liaquat National Hospital and Medical College, Karachi, PAK; 3 Internal Medicine, Baqai Medical University, Karachi, PAK; 4 Statistics, Liaquat National Hospital and Medical College, Karachi, PAK; 5 Pathology, Multan Medical and Dental College, Multan, PAK

**Keywords:** liquid-based cytology, conventional papanicolaou (pap) smear, cervical cancer screening

## Abstract

Introduction

After the introduction of cervical cancer screening program with cervical cytology, a marked decline in deaths secondary to cervical cancer was observed in developed countries. Two methods are used for cervical cytology. The first one is the conventional Papanicolaou (PAP) and the second one is liquid-based cytology (LBC). Although various studies in western countries established the role of LBC in cervical cancer screening, no large-scale study was conducted in our population to compare the two techniques for cervical cancer screening. Therefore, in this study, we compared the diagnostic utility of these two techniques for detecting cervical epithelial lesions.

Methods

A total of 3,929 patients, who presented to the Gynecology Clinic, Liaquat National Hospital, for cervical cancer screening from January 2015 until December 2019, over a period of five years, were included in the study. A total of 1,503 specimens were prepared by LBC, and 2,426 specimens were prepared by a conventional PAP smear method. All smears were interpreted using the Bethesda System of Reporting Cytopathology.

Results

The mean age of the patients was 39.46±11.14 years. For cytological evaluation, 98.7% of specimens were adequate. The inadequacy rate was 1.3% for conventional PAP smear and 1.2% for LBC. While 97.2% of specimens were reported as negative for intraepithelial lesion or malignancy, 1.1% of specimens showed squamous epithelial lesions. There was a significant difference in the detection rate of squamous epithelial lesions using the two techniques. The detection rate of squamous intraepithelial lesions using LBC was 2.1%, which was higher than that of the conventional PAP smear (0.6%). The detection rates of glandular lesions using LBC and conventional PAP smear were 0.5% and 0.2%, respectively.

Conclusion

We found a higher disease detection rate of squamous epithelial lesions using LBC compared to conventional PAP smear. Therefore, we recommend a widespread use of LBC for mass cervical cancer screening in our population.

## Introduction

Gynecological cancers are a major source of morbidity and mortality among women [1-3]. After the introduction of cervical cancer screening program with cervical cytology and high-risk human papillomavirus (hrHPV) testing, a marked decline in deaths secondary to cervical cancer was observed in developed countries [4]. Two methods are used for cervical cytology. First method is the conventional Papanicolaou (PAP) smear, which is still the most commonly used method for cervical cancer screening in Pakistan, as it is cheap and widely available. Second method is the liquid-based cytology (LBC); although it is the most familiar method of cervical cancer screening in western countries, it is only available at a few facilities in Pakistan. Although conventional PAP smear is cheap, it is limited due to sampling and interpretive issues. A lot of cellular material is wasted during sample collection as a result of two reasons. First, the cells are not properly collected on the sampling device from the patient, and, second, cells are not adequately transferred from the sampling device onto the slide. In addition, abnormal cells present on the slide are misinterpreted due to hemorrhagic or inflammatory background, resulting in misinterpretation. Alternatively, LBC leads to a thin monolayered smear with a clear background. In addition, cellular material is preserved for future studies, if needed. Although various studies in western countries established the role of LBC in cervical cancer screening, no large-scale study was conducted in our population to compare the two techniques for cervical cancer screening. Therefore, in this study, we compared the diagnostic utility of these two techniques for detecting cervical epithelial lesions.

## Materials and methods

**Figure 1:**
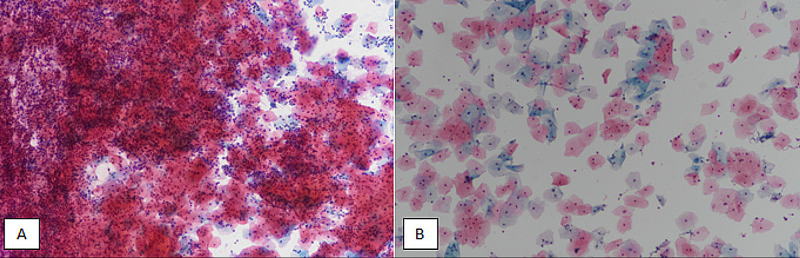
Comparison of conventional PAP smear and LBC for cervical cytology. (A) Conventional PAP smear at 100X magnification showing overlapping squamous cells with a lot of inflammatory and hemorrhagic background, making individual cellular details difficult to interpret. (B) LBC at 100X magnification depicting widely dispersed squamous cells without any inflammation or hemorrhage in the background, resulting in clear individual cellular details. PAP, Papanicolaou; LBC, liquid-based cytology

A total of 3,929 patients, who presented to the Gynecology Clinic, Liaquat National Hospital (LNH), for cervical cancer screening from January 2015 until December 2019, over a period of five years, were included in the study. Known cases of gynecological cancers were excluded from the study. The specimens were collected in the gynecology clinic. The procedures were performed by gynecology fellows. The specimens obtained were then transferred to the Cytopathology Department, LNH. A total of 1,503 specimens were prepared using LBC, and 2,426 specimens were prepared using a conventional PAP smear method. A comparison of smears prepared using LBC and conventional PAP smear is shown in Figure 1.

For conventional PAP smear, the samples were collected with a spatula and a brush, which were then smeared on a slide and stained with PAP stain. For LBC, samples were collected with a brush, which were then thoroughly rinsed in a preservation vial. The vial was then processed by an automated machine (thin-prep processor). The percentage of inadequacy and the disease detection rates were compared between specimens prepared using LBC and conventional PAP smear methods. The slides were first screened by the cytotechnologists and then reported by the consultant cytopathologists. All smears were interpreted using the Bethesda System of Reporting Cytopathology.

## Results

The mean age of the patients was 39.46±11.14 years. For cytological evaluation, 98.7% of specimens were adequate. The inadequacy rate was 1.3% for conventional PAP smear and 1.2% for LBC. While 97.2% of specimens were reported as negative for intraepithelial lesion or malignancy (NILM), 1.1% of specimens showed squamous epithelial lesions. There was a significant difference in the detection rate of squamous epithelial lesions using the two techniques. The detection rate of squamous epithelial lesions using LBC was 2.1%, which was higher than that of the conventional PAP smear (0.6%). The detection rates of glandular lesions using LBC and conventional PAP smear were 0.5% and 0.2%, respectively. Comparison of LBC and conventional PAP smear in terms of diagnostic categories is presented in Table 1.

**Table 1:** Comparison of liquid-based cytology and conventional PAP smear in terms of diagnostic categories Chi-square test was applied. *Significant at <0.05. PAP, Papanicolaou; LBC, liquid-based cytology; NILM, negative for intraepithelial lesion or malignancy

Cytological diagnostic category	Frequency (%)			p-Value
	Conventional PAP smear (n = 2426)	LBC (n = 1503)	Total (n = 3929)	
Inadequate	32 (1.3)	18 (1.2)	50 (1.3)	<0.0001*
NILM	2374 (97.9)	1446 (96.2)	3820 (97.2)	
Squamous epithelial lesions	14 (0.6)	31 (2.1)	45 (1.1)	
Glandular epithelial lesions	6 (0.2)	8 (0.5)	14 (0.4)	

Table 2 shows the comparison of LBC and PAP smears in terms of individual diagnoses. Among infectious/reactive conditions, bacterial vaginosis, candidiasis, and radiation changes were noted in 2.1%, 4.5%, and 0.2% cases, respectively. The detection rate of atrophic vaginitis was 19% using LBC, whereas it was 15.3% using conventional PAP smear. The disease detection rates of low-grade squamous intraepithelial lesions (LSILs) and high-grade squamous intraepithelial lesions (HSILs) were 0.7% and 0.4%, respectively, using LBC, compared to 0.04% and 0.2%, respectively, using conventional PAP smear. The detection rates of atypical squamous cells of undetermined significance (ASC-US) were 1% and 0.4% using LBC and conventional PAP smear, respectively. 

**Table 2:** Comparison of LBC and conventional PAP smear in terms of individual diagnoses Fisher's exact test was applied. *Significant at <0.05. PAP, Papanicolaou; LBC, liquid-based cytology; ASC-US, atypical squamous cells of undetermined significance; LSIL, low-grade squamous intraepithelial lesion; HSIL, high-grade squamous intraepithelial lesion; AGUS, atypical glandular cells; NILM, negative for intraepithelial lesion or malignancy; NOS, not otherwise specified

Cytological diagnosis	Frequency (%)			p-Value
	Conventional PAP smear (n = 2426)	LBC (n = 1503)	Total (n = 3929)	
Inadequate	32 (1.3)	18 (1.2)	50 (1.3)	<0.0001*
Candidiasis	112 (4.6)	64 (4.3)	176 (4.5)	
Bacterial vaginosis	45 (1.9)	36 (2.4)	81 (2.1)	
Trichomoniasis	3 (0.1)	5 (0.3)	8 (0.2)	
Actinomycosis	1 (0.04)	0 (0)	1 (0.025)	
Viral changes (herpes simplex)	1 (0.04)	0 (0)	1 (0.025)	
Atrophic vaginitis	371 (15.3)	286 (19)	657 (16.7)	
Endometrial cells	17 (0.7)	9 (0.6)	26 (0.7)	
Reactive changes	25 (1.0)	7 (0.5)	32 (0.8)	
Radiation changes	4 (0.2)	3 (0.2)	7 (0.2)	
Nonspecific inflammation	669 (27.6)	109 (7.3)	778 (19.8)	
ASC-US	9 (0.4)	15 (1)	24 (0.6)	
LSIL	1 (0.04)	10 (0.7)	11 (0.3)	
HSIL	4 (0.2)	6 (0.4)	10 (0.3)	
AGUS, favor neoplastic	6 (0.2)	7 (0.5)	13 (0.3)	
Adenocarcinoma	0 (0)	1 (0.1)	1 (0.025)	
NILM (NOS)	1126 (46.4)	927 (61.7)	2053 (52.3)	

## Discussion

In this study, we found that the disease detection rate of squamous epithelial lesions was higher when using LBC compared to the conventional PAP smear; however, no significant difference was seen in terms of specimen adequacy. In addition, we also found a slightly increased detection rate of glandular lesions and infections, such as bacterial vaginosis and trichomoniasis, when using LBC compared to the conventional PAP smear. 

**Figure 2:**
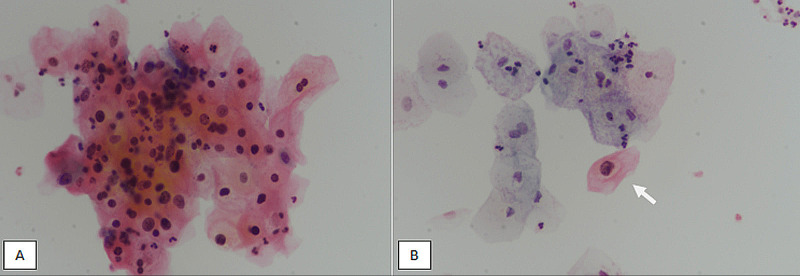
Low-grade squamous intraepithelial lesion. (A) LBC at 400X magnification revealing a cluster of atypical squamous cells showing nuclear enlargement, hyperchromasia, and anisonucleosis. (B) LBC at 400X magnification showing an atypical large squamous cell with intermediate squamous cell type cytoplasm (arrow). The atypical cell has a nucleus greater than three times the size of an intermediate squamous cell. It also shows koilocytic change (perinuclear cavitation) (arrow). LBC, liquid-based cytology

We used the the Bethesda System of Reporting Cytopathology in our study. The Bethesda system divides squamous cell abnormalities into ASC-US, atypical squamous cells (cannot exclude HSIL [ASC-H]), LSIL, HSIL, and squamous cell carcinoma (SCC). ASC-US and ASC-H are ambiguous categories requiring repeat smear after adequate time interval, whereas LSIL, HSIL, and SCC require further evaluation by hrHPV testing or cervical biopsy. The cytological diagnosis of LSIL is characterized by large squamous cells (similar to intermediate cells) showing nuclear enlargement (three times the size of an intermediate cell nucleus), along with nuclear hyperchromasia, anisonucleosis, and perinuclear cavitation (koilocytosis) (Figure 2).

Follow-up studies after the diagnosis of LSIL by cervical cytology reported disease progression in 0.7% women, whereas persistent lesions were noted in 10.8% women [5].

**Figure 3:**
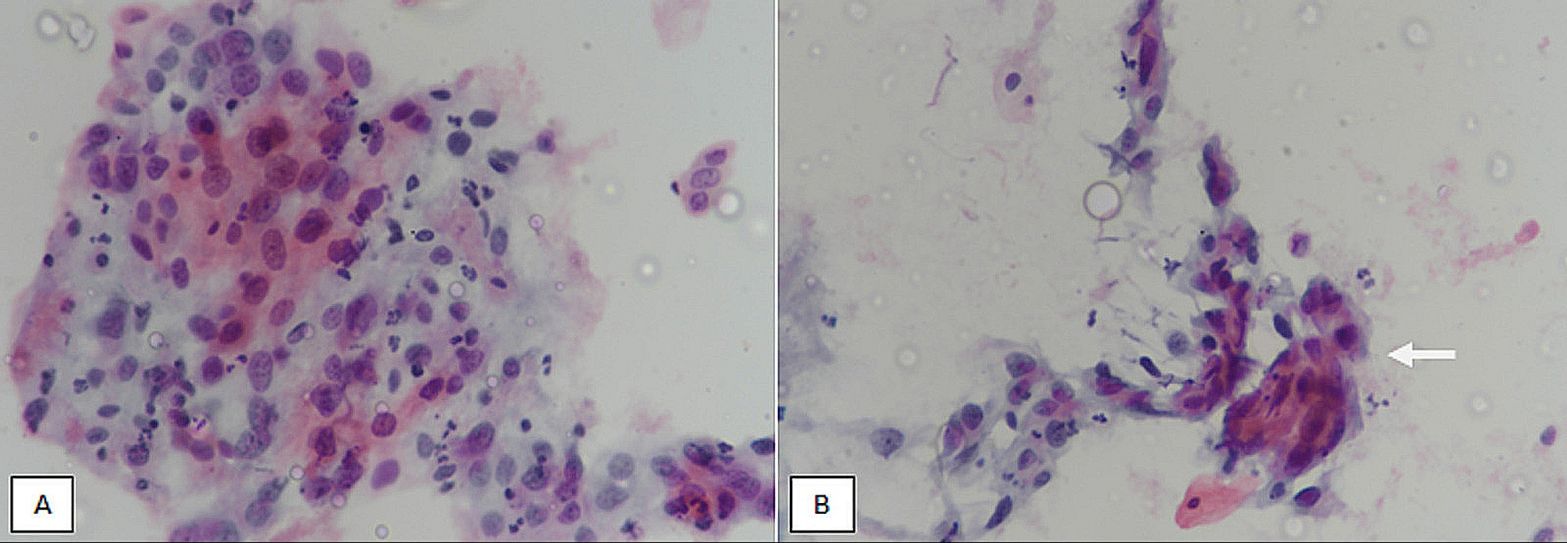
High-grade squamous intraepithelial lesion. (A) LBC at 400X magnification showing syncytial-like aggregates of atypical squamous cells with marked nuclear atypia. (B) LBC at 400X magnification showing hyperchromatic crowded groups of slightly smaller squamous cells (compared to low-grade squamous intraepithelial lesions). Cells show high nuclear-to-cytoplasmic ratio and irregular hyperchromatic nuclei (arrow). LBC, liquid-based cytology

HSIL is characterized by smaller squamous cells (compared to LSIL) that are present singly or in syncytial groups with higher nuclear-to-cytoplasmic ratio and more pronounced nuclear abnormalities compared to LSIL in terms of hyperchromasia and nuclear contour irregularities (Figure 3).

**Figure 4:**
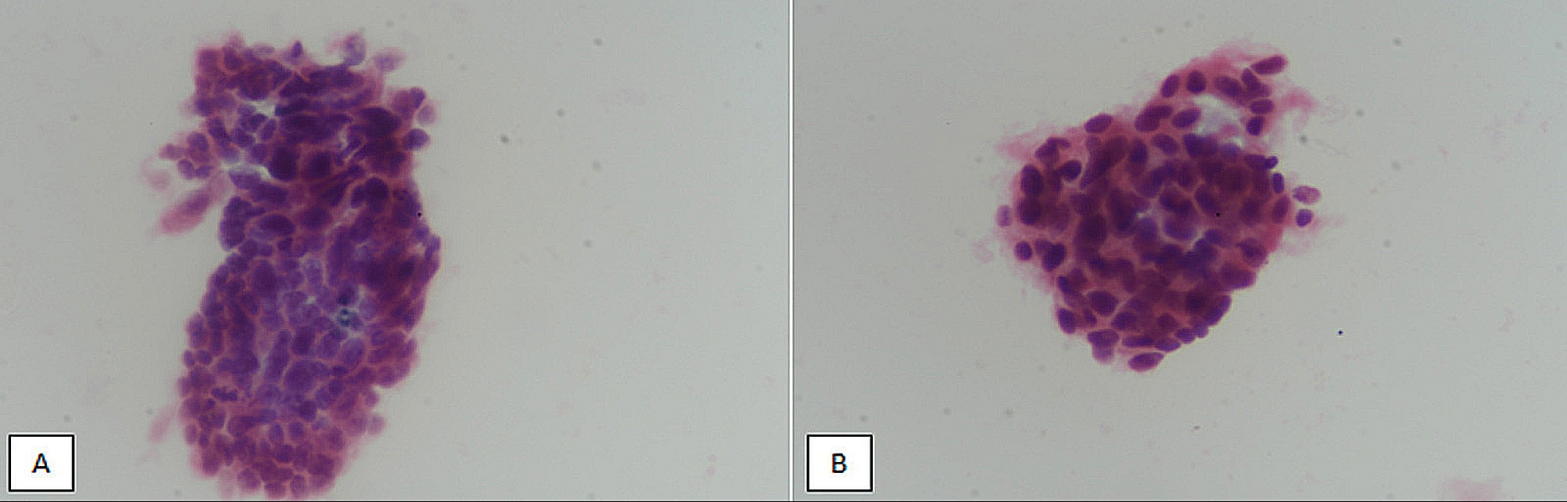
Adenocarcinoma (endocervical). (A, B) Liquid-based cytology at 400X magnification revealing atypical clusters of cells with glandular configuration showing hyperchromasia and pseudostratification.

Glandular cell abnormalities include atypical glandular cells of undetermined significance (AGUS), adenocarcinoma in situ, and adenocarcinoma. The diagnosis of adenocarcinoma on cytology requires the presence of the group of cells in clusters, pseudostratified strips, or rosettes with chromatin abnormalities and background tumor diathesis (may not be present in smears prepared using the LBC method) (Figure 4).

In our study, the detection rate of glandular epithelial lesions was 0.5% using LBC. Studies have shown that unlike squamous epithelial lesions (that can regress with time), AGUS is associated with a persistent risk of cervical cancer (cervical adenocarcinoma) [6].

A study conducted in Japan examined the advantages of LBC over conventional PAP smear in mass screening for cervical cancer. They reported that the detection rate of ASC-US and more severe lesions using LBC was 1.44%, whereas it was 1.13% using conventional PAP smear, hence a 1.3-fold higher detection rate when using LBC [7]. Our results showed comparable findings. Another study involving 310 cases compared conventional PAP smear with LBC and reported a significant difference in the inadequacy rate between the two methods. Unsatisfactory smears were noted to be 7.1% in the conventional PAP smear compared to 1.6% in cases by LBC. They did not find any significant difference in the disease detection rate [8]. Conversely, we found a significant difference in the disease detection rate between conventional PAP smear and LBC. Similarly, a prospective study including 312 cases of abnormal cervical cytology concluded that although LBC had a higher sensitivity of detecting epithelial lesions, overall there was no significant difference in the performance of LBC compared with conventional PAP smear [9].

There were a few limitations of our study. First, the diagnostic accuracy of LBC was not evaluated in our study as biopsy results were not available to calculate the sensitivity and specificity of LBC with respect to histology. Second, hrHPV testing was not performed to determine the prevalence of hrHPV in patients with squamous and glandular lesions on LBC. Therefore, we recommend large-scale prospective studies to determine the diagnostic accuracy of LBC along with the prevalence of hrHPV in patients with glandular and squamous lesions on LBC to have a better insight into the utility of LBC for cervical cancer screening in our population. 

## Conclusions

In our study, it was documented that the disease detection rate for squamous epithelial abnormalities was significantly higher using LBC than the conventional PAP smear. In addition, LBC has several other advantages over PAP smear as the material can be used in molecular studies, such as the detection of hrHPV. Moreover, in the long run, LBC is also cost-effective in mass cervical cancer screening, as LBC is needed less frequently than the conventional PAP smear. Therefore, we recommend widespread use of LBC for cervical cancer screening in our population.

## References

[1] Hashmi AA, Hussain ZF, Irfan M (2019). Epidermal growth factor receptor (EGFR) overexpression in endometrial carcinoma: association with histopathologic parameters. Surg and Exp Pathol.

[2] Hashmi AA, Iftikhar SN, Ali J, Shaheen F, Afroze F, Imran A (2020). Morphological spectrum and pathological parameters of type 2 endometrial carcinoma: a comparison with type 1 endometrial cancers. Cureus.

[3] Hashmi AA, Hussain ZF, Qadri A (2018). Androgen receptor expression in endometrial carcinoma and its correlation with clinicopathologic features. BMC Res Notes.

[4] US Preventive Services Task Force (2018). Screening for cervical cancer: US Preventive Services Task Force recommendation statement. JAMA.

[5] Ciavattini A, Clemente N, Tsiroglou D (2017). Follow up in women with biopsy diagnosis of cervical low-grade squamous intraepithelial lesion (LSIL): how long should it be?. Arch Gynecol Obstet.

[6] Wang J, Andrae B, Sundström K (2016). Risk of invasive cervical cancer after atypical glandular cells in cervical screening: nationwide cohort study. BMJ.

[7] Yokoyama Y, Futagami M, Watanabe J, Sakuraba A, Nagasawa K, Maruyama H, Sato S (2016). The advantages of incorporating liquid-based cytology (TACAS™) in mass screening for cervical cancer. Hum Cell.

[8] Pankaj S, Nazneen S, Kumari S (2018). Comparison of conventional Pap smear and liquid-based cytology: a study of cervical cancer screening at a tertiary care center in Bihar. Indian J Cancer.

[9] Nishio H, Iwata T, Nomura H (2018). Liquid-based cytology versus conventional cytology for detection of uterine cervical lesions: a prospective observational study. Jpn J Clin Oncol.

